# Phytosterol‐based edible oleogels: A novel way of replacing saturated fat in food

**DOI:** 10.1111/nbu.12325

**Published:** 2018-05-08

**Authors:** A. Matheson, G. Dalkas, P. S. Clegg, S. R. Euston

**Affiliations:** ^1^ School of Physics and Astronomy University of Edinburgh Edinburgh UK; ^2^ School of Engineering and Physical Sciences Heriot‐Watt University Edinburgh UK

**Keywords:** oleogels, phytosterols, β‐sitosterol, γ‐oryzanol

## Abstract

This article presents a summary of recent results relating to phytosterol oleogels. Oleogels represent a novel way of replacing saturated fat in food, whilst phytosterols have been shown to actively lower low‐density lipoprotein (LDL)‐ cholesterol levels. There are a number of technical challenges to exploiting phytosterol oleogels, including a high sensitivity to water. To facilitate their incorporation into food, the fundamental physiochemical processes which mediate the formation of these gels and two different approaches to produce phytosterol oleogels that are stable in the presence of water were explored as part of the recent Biotechnology and Biological Sciences Research Council (BBSRC)–Diet and Health Research Industry Club (DRINC)–funded *Edible Oleogels for Reduction of Saturated Fat* project. This report summarises the findings, which will support the development of healthier food products that are lower in saturated fat and acceptable to consumers.

## Introduction

One of the biggest societal challenges in the twenty‐first century is obesity and related health conditions, which are projected to cost the NHS approximately £2 billion annually by 2030 (Wang *et al*. 2011). This problem is not just isolated to developed economies, with obesity rates also increasing rapidly in countries such as India and China (Kumanyika *et al*. 2002). The prevalence of obesity worldwide has increased over a number of decades due to a range of factors, making it difficult to offer a single solution. However, given that this rise has coincided with an increase in processed or pre‐prepared food consumption, and that these types of foods are often high in fat, salt and sugars and make up a substantial proportion of the current UK diet (Monteiro *et al*. 2017), the reformulation of these foods could play a role. In many of these foods, solid fats are used in part to structure the food. They ensure the food has the correct form, texture and is shelf‐stable. Fats are structured via the inclusion of triglyceride (TAG) molecules (three fatty acids esterified to a glycerol backbone) that crystallise, forming fat crystals. The food industry is using hydrogenation, interesterification and fractionation methods for the production of solid fats with vegetable oil (Mills *et al*. 2017). The fabricated solid fats, which are typically used in breakfast, bakery and chocolate products are associated with high saturated fat content. The consumption of some saturated (particularly animal) fats increases the risk of cardiovascular disease (CVD) by raising the levels of low‐density lipoprotein (LDL)‐cholesterol (bad cholesterol), in contrast to mono‐ and polyunsaturated fats which tend to lower LDL‐cholesterol levels and can result in weight gain due to its high energy density (Mensink 2016). Although there is ongoing debate on the impact of saturated fats on CVD risk (Bier 2016), a report of the joint World Health Organization (WHO)/Food and Agriculture Organization (FAO) expert consultation on *Diet, Nutrition and the Prevention of Chronic Diseases* (WHO 2003), the recently released by the US Department of Health and Human Services (HHS) and the US Department of Agriculture (USDA) 2015–2020 *Dietary Guidelines for Americans* report that the risk of developing non‐communicable diseases (NCDs) is lowered by decreasing saturated fat intake to <10% of total energy and replacing with unsaturated fats. Not only is the intake of saturated fat detrimental to the health of the population [coronary heart disease remains the biggest cause of mortality worldwide (WHO 2017)], but it also has a huge associated economic cost. The Centre for Economics and Business Research has estimated the economic burden of CVD across six European economies (France, Germany, Italy, Spain, Sweden and the UK) at over €102 billion in 2014, which is predicted to increase to €122.6 billion by 2020 (CEBR 2014).

A major difficulty for food manufacturers is to remove saturated fat from a food formulation as this often contributes to the solid texture in ways that unsaturated liquid oils cannot, thus altering the sensorial characteristics. One possible means of reducing saturated fat and improving the nutritional profile and energy content of food products is to replace it with edible oleogels. Edible oleogels (also commonly referred to as organogels) are liquid oils structured and solidified/gelled by non‐triglyceride networks, and thus take on solid‐like properties.

Oleogelation for food structuring has received a great deal of interest from academic researchers and industrial scientists. Oleogelation of liquid oils can be achieved using, as building blocks, oleogelators, such as phytosterols, phospholipids, vegetable waxes or mono‐ and diglycerides, which structure into a 3D network that can trap oil. Research with phytosterols suggests that edible oleogels made with these compounds could have LDL‐cholesterol lowering effects. For example, studies have shown that phytosterols at dosages of 2–3 g/day lower LDL‐cholesterol levels by 6%–15% (NCEP 2002).

Gelation may be achieved via a range of different routes, but always involves the introduction of a fluid spanning network which arrests the flow of the oil. The composition of the oleogel will differ depending on the means of gelation, and by changing the concentration of gelator in the system it is possible to tune properties such as the hardness and melting point of the gel to suit the desired application. Therefore, oleogels may represent a means of replacing solid saturated fats with oleogelled liquid unsaturated fats that are considered healthier, without significantly compromising the sensorial properties of the food. For example, in a recent study by Panagiotopoulou *et al*. (2016), a phytosterol‐based oleogel was used to replace up to half of added pork back fat (10% out of the total 20% added) in frankfurter sausages. They demonstrated that several sensorial attributes, such as appearance, colour, smell, taste, texture, oiliness and juiciness, as evaluated by a trained panel, did not significantly affect the overall sensorial properties, nor the experimentally measured texture profile.

**Figure 1:**
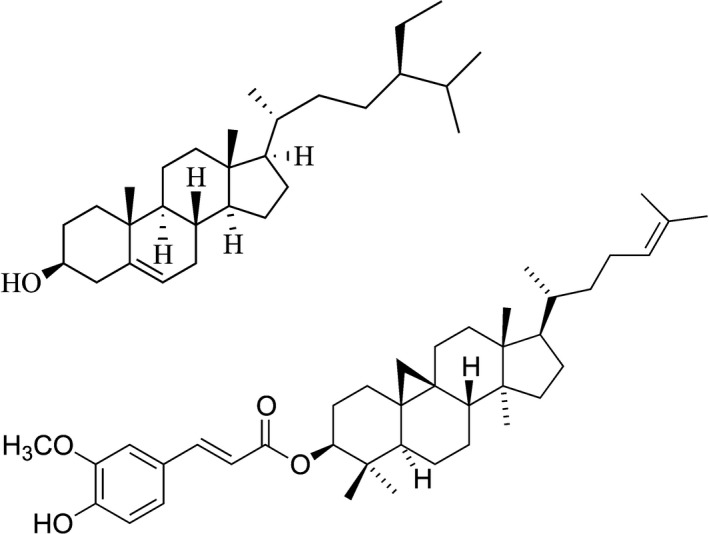
Structures of β‐sitosterol (top) and γ‐oryzanol (bottom).

One of the most appealing edible oleogel systems is that of the phytosterol β‐sitosterol and the phytosterol ester γ‐oryzanol (structures shown in Fig. 1). Phytosterols have been shown to lower blood cholesterol levels (Katan *et al*. 2003), thus raising the possibility of an organogel which can be used to simultaneously reduce saturated fat intake and introduce an active ingredient which offers further health benefits. It has, however, proven difficult to formulate these phytosterols into an oleogel product. This is due in part to the sensitivity of the oleogel to the presence of even small quantities of water, and exacerbated by the lack of availability of alternative phytosterol esters to replace γ‐oryzanol. Due to this, a better understanding is needed of the structure of these gels to help identify other novel oleogels and oleogelating phytosterols.

## Results of the ‘Edible Oleogels for Reduction of Saturated Fat’ project

**Figure 2:**
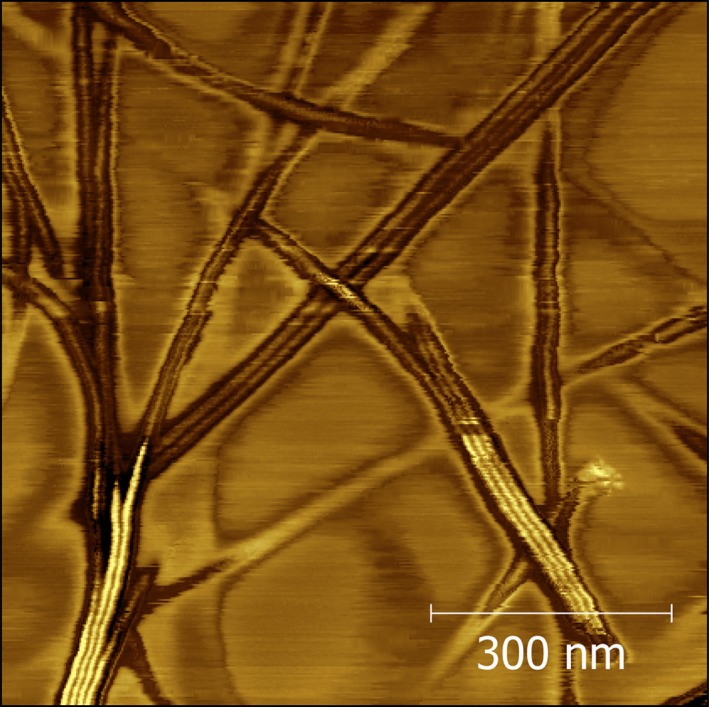
Phase mode atomic force microscopy (AFM) image of a sitosterol–oryzanol in sunflower oil gel. Source: Matheson *et al*. (2017aa,b) reprinted with permission from the American Chemical Society. [Colour figure can be viewed at http://wileyonlinelibrary.com]

The Biotechnology and Biological Sciences Research Council (BBSRC) Diet and Health Research Industry Club (DRINC)–funded *Edible Oleogels for Reduction of Saturated Fat* project began by imaging oleogels using atomic force microscopy (AFM), as data from previous attempts of imaging these gels using this approach were very limited. The images obtained offered unparalleled resolution of the oleogel network as shown in Fig. 2. These images are consistent with previous small angle neutron scattering (SANS) results, which suggested sitosterol and oryzanol arrange themselves in a helical fashion to create a hollow tubule with ~10 nm diameter (Bot *et al*. 2012). The tubules in Fig. 2 are evident, and it was observed that they adhere to each other to form bundles that split apart at intervals to make a complex, bifurcating fibril network that forms the structure that traps oil and gives the oleogel its solid texture.

**Figure 3:**
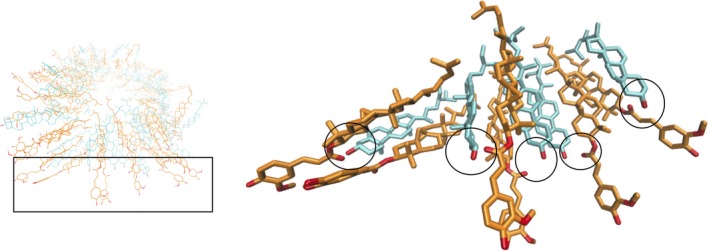
Molecular dynamics simulations of a sitosterol and oryzanol tubule. The image on the right is a zoomed in image of the region shown in the black box on the left. Intermolecular hydrogen bonds are outlined with black rings. Taken from Matheson *et al*. (2018) – published by The Royal Society of Chemistry. [Colour figure can be viewed at http://wileyonlinelibrary.com]

Having confirmed the presence of these 10 nm fibrils as the building blocks of the gel, research was conducted to understand why they form using computer models of fibril structure using molecular dynamics (MD) simulations. MD simulation is a technique that allows the dynamics of molecules at the molecular level to be investigated in a way that is difficult or impossible to replicate experimentally. It is necessary to make some approximations for the interactions between the atoms in the simulated system, which means that MD is at its most useful when combined with confirmatory experiments, as was the case in this BBSRC‐DRINC project. Molecules were arranged into a tubule as predicted by neutron scattering measurements (Bot *et al*. 2012). A cross section of the tubule is shown in the left panel of Fig. 3, with a zoomed in portion shown on the right. The formation of hydrogen bonds between the hydroxyl group of the sitosterol and the carbonyl group of the oryzanol were observed, as previously suggested by molecular energy minimisation calculation and Fourier transform infrared (FTIR) spectroscopy (Pernetti *et al*. 2007; Bot *et al*. 2012), with these interactions forming a cooperative network that stabilises the fibril.

Whilst sitosterol–oryzanol gels have been shown to be acceptable to consumers when incorporated into frankfurter sausages (Panagiotopoulou *et al*. 2016), they have not been employed widely to structure foods such as margarines and spreads where the solid fat constitutes the majority of the structure. This is partly due to the inherent instability of the gels in the presence of water found in water‐in‐oil margarine and spread formulations. The mechanism for this instability is the tendency for water to bond to the hydroxyl group of the sitosterol, promoting the formation of hydrate crystals and breaking up the hydrogen bonded network illustrated in Fig. 3. It has been shown that by reducing the water activity of the aqueous phase with salt or sugar, the effect of water may be mitigated somewhat (Sawalha *et al*. 2012). Presumably, a similar effect comes into play with the frankfurters formulated by Panagiotopoulou *et al*. (2016), where the presence of proteins and hydrocolloids (starch) modify water activity to a degree sufficient to allow the oleogel to remain stable. An alternative approach is to make emulsions where the water is replaced with an alternative polar phase. Glycerol was selected as it is food safe, cheap and non‐volatile. Additionally, it is commonly used as a sweetener, so any oleogel‐glycerol emulsions may be well suited to low‐fat confectionary applications.

**Figure 4:**
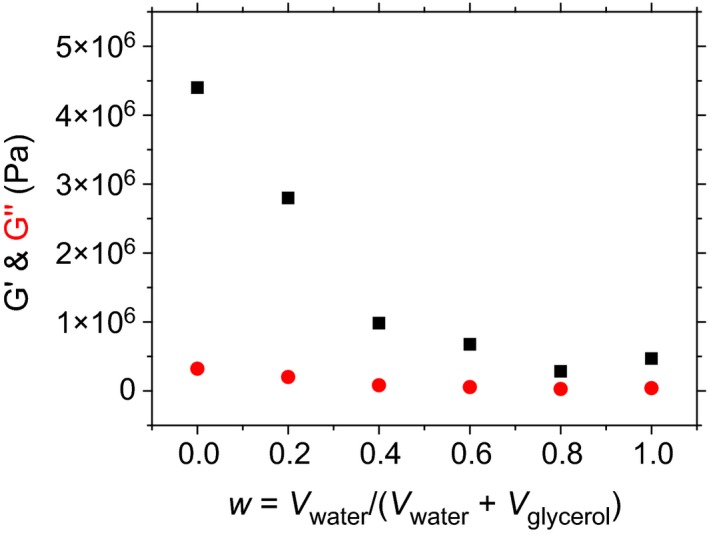
G′ (black squares) and G″ (red circles) as a function of water content added to form organogel emulsions. G′ is the storage modulus, a measure of how ‘solid‐like’ the response of the gel to shear is. G″ is the loss modulus, a measure of how ‘liquid‐like’ the response of the gel to shear is. Taken from Matheson *et al*. (2018) – published by The Royal Society of Chemistry. [Colour figure can be viewed at http://wileyonlinelibrary.com]

**Figure 5:**
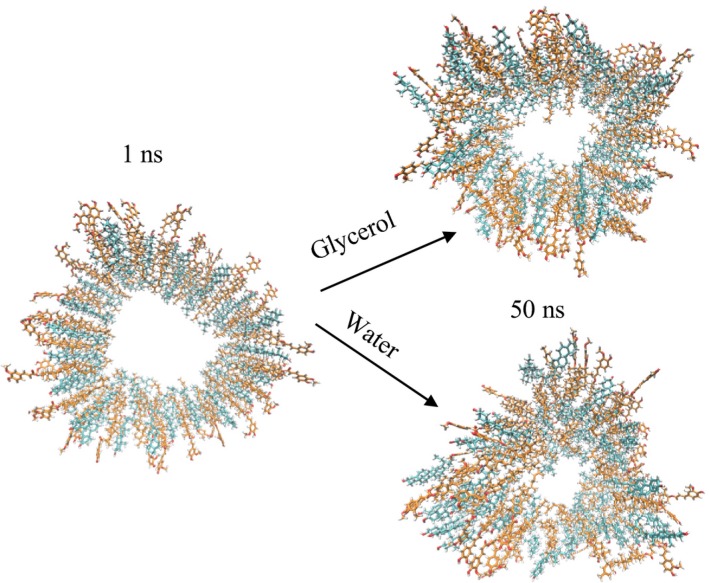
Cross section of a sitosterol–oryzanol fibril, and its confirmation after 50 ns immersed in glycerol (top) and water (bottom). The fibrils exhibit a higher stability (more phytosterol interactions and less disruption to fibril structure) in glycerol than in water, and therefore were more stable in the former. Figure taken from Matheson *et al*. (2018) – published by The Royal Society of Chemistry. [Colour figure can be viewed at http://wileyonlinelibrary.com]

Shown in Fig. 4 is how the solidity of the gel sample (indicated by the G′ or elastic modulus values) of sitosterol–oryzanol oleogel emulsions varies with the composition of the polar phase from pure glycerol (w = 0) to pure water (w = 1). This is a good indication that glycerol is less damaging to the oleogel network than water. This was confirmed by molecular dynamics simulations, where it was observed that sitosterol–oryzanol tubules are more stable in the presence of glycerol than water, as demonstrated in Fig. 5.

Another interesting observation was that highly stable multiple emulsions could be formed when dispersing glycerol through the oleogel network. These could have applications in delayed release nutraceuticals or pharmaceuticals.

As outlined earlier, there are numerous methods for producing organogels, including self‐assembled fibrillar networks such as those formed by sitosterol and oryzanol. The next phase of the project explored whether another type of oleogel could be formed that incorporated phytosterols, due to their health‐giving properties. Phytosterols fall into the larger family of bio‐molecules known as steroids, along with bile acids and their salts. Bile acids and bile salts have been shown to form oleogels when mixed at the correct molar ratio with lecithin, a commonly used emulsifier in foods (Tung *et al*. 2006; Njauw *et al*. 2013). Given the similarity in structure between these molecules and oryzanol, whether oryzanol would form oleogels with lecithin was explored. Lecithin and oryzanol were found to interact strongly, with lecithin solubilising oryzanol in sunflower oil at concentrations it would normally precipitate at, and vice versa (Matheson *et al*. 2017a,b). A combination of light scattering, spectroscopy and molecular dynamics simulations indicates that the mechanism for this is the formation of mixed micelles, whereby the oryzanol intercalates between lecithin molecules. However, although there was a modest increase in the viscosity of the system, it was not as marked as that observed with the addition of bile salts to lecithin dispersions (Tung *et al*. 2006; Njauw *et al*. 2013), which is associated with a transition from spherical to highly elongated, wormlike micelles. Oryzanol appears to sit between the lecithin molecules in such a manner that the packing ratio of lecithin molecules is not significantly altered, and thus, the micelles remain spherical.

Despite this, it was possible to form solid‐like gels with lecithin and oryzanol by adding water. As previously explained, the addition of water is highly deleterious to sitosterol–oryzanol oleogels. However, the mechanism for this is the formation of hydrate crystals between sitosterol and water. Due to the absence of sitosterol from the oryzanol‐lecithin system, this does not occur. Instead, the water appears to be sequestered into the micelles, which then stick together due to van der Waals forces, providing the gel with a yield stress and solid‐like mechanical properties. It is therefore possible to produce an oleogel containing phytosterols, which also contain a similar water content and viscoelastic properties to low‐fat spreads.

## Concluding remarks

Oleogels are one of the most attractive current systems for adoption in food, considering that there is increasing demand for solid fat replacers. Oleogels have the potential to be used in products such as chocolate, dough and pastry, spreads, processed meat products and ice cream, although further work is needed to translate the laboratory experiments to factory scale. The number of filed patents for oleogels has increased in the last decade, indicating the potential commercial value of such applications. Because of this potential, researchers in academia and the food industry are trying to find new molecules that can act as oil gelling structurants. The results from the *Edible Oleogels for Reduction of Saturated Fat* project represent only a small portion of the wide range of research currently being undertaken on oleogels.

There are some challenges that should be addressed in order to achieve marketable consumer products containing oleogels. The complexity of gel formation and interactions between gelator molecules makes it difficult to predict which gelators will work for any particular edible oils (Hughes *et al*. 2009). Further research on the formation and structure of oleogels will lead to a better understanding of these systems and perhaps lead to the discovery of the most suitable gelator of edible oils for a particular application. There should be no major safety concerns with oleogels, as phytosterols, oryzanol and sitosterol are found naturally in foods and neither have any significant cytotoxic effects (Moon *et al*. 2017; Paniagua‐Pérez *et al*. 2005) and are used in foods already. However, other potential gelators may not have the required approval to be used as direct ingredients in foods in the amounts that are necessary to form an organogel; therefore, certain regulatory changes will be needed to enable this process. Importantly, the major challenges are still the ability of oleogels to mimic the mouthfeel and texture of commonly used saturated fats, and the sensitivity of the phytosterol oleogels to water. For phytosterol‐based oleogels, the challenge is to formulate water stability into the system so it can be used for spreads and margarine type products, as this is where the greatest impact on saturated fat reduction is likely to be. Currently, the successful applications have been where water activity is controlled through interaction with other ingredients. Proteins and hydrocolloids, such as starch, are not usually added to spreads and margarines so the water phase tends to be more disruptive to fibril stability. To apply this phytosterol oleogelling technology to spreads and margarines, other ways to control water–tubule interactions, such as the lecithin and glycerol containing oleogels reported here, will need to be identified. Alternatively, a better understanding of the interactions and physicochemical factors that control tubule stability may help to identify new oleogelating phytosterol combinations that are more stable to the presence of water. It is hoped that the results from the demonstrate the valuable role physical sciences play in the development of novel ingredients and the reformulation of foods, for the delivery of healthier products that are acceptable to consumers.

## Conflict of interest

The authors have no conflict of interest to declare.
